# Characteristics, outcomes, facilitators and barriers for psychosocial interventions on inpatient mental health dementia wards: a systematic review

**DOI:** 10.1186/s12877-024-04965-8

**Published:** 2024-04-23

**Authors:** Naomi Thompson, Ming-Hung Hsu, Helen Odell-Miller, Benjamin R. Underwood, Emma Wolverson

**Affiliations:** 1 Cambridge Institute for Music Therapy Research, Anglia Ruskin University, Cambridge, UK; 2 Arts Therapies Services, Cambridgeshire and Peterborough NHS Foundation Trust, Fulbourn Hospital, Fulbourn, UK; 3 Department of Psychiatry, University of Cambridge, Cambridge, UK; 4 Cambridgeshire and Peterborough NHS Foundation Trust, Fulbourn Hospital, Fulbourn, UK; 5 Faculty of Science and Engineering, Anglia Ruskin University, Cambridge, UK; 6 Dementia UK, London, UK; 7 Faculty of Health Sciences, University of Hull, Hull, UK

**Keywords:** Psychosocial interventions, Inpatient dementia care, Mental health, Systematic review

## Abstract

**Background:**

The National Institute for Health and Care Excellence guidelines state that psychosocial interventions should be the first line of treatment for people with dementia who are experiencing distress behaviours, such as agitation and depression. However, little is known about the characteristics and outcomes of psychosocial interventions or the facilitators and barriers to implementation on inpatient mental health dementia wards which provide care for people with dementia who are often experiencing high levels of distress.

**Methods:**

A systematic search was conducted on MEDLINE, CINAHL, PsycINFO, Psychology and Behavioural Sciences Collection, and Scopus in May 2023, following PRISMA guidelines. Reference and citation searches were conducted on included articles. Peer-reviewed literature of any study design, relating to psychosocial interventions in inpatient mental health dementia wards, was included. One author reviewed all articles, with a third of results reviewed independently by a second author. Data were extracted to a bespoke form and synthesised using a narrative review. The quality of included studies was appraised using the Mixed Methods Appraisal Tool.

**Results:**

Sixteen studies were included in the synthesis, which together included a total of 538 people with dementia. Study methods and quality varied. Psychosocial interventions delivered on wards included music therapy (five studies), multisensory interventions (four studies), multicomponent interventions (two studies), technology-based interventions (two studies), massage interventions (two studies) and physical exercise (one study). Reduction in distress and improvement in wellbeing was demonstrated inconsistently across studies. Delivering interventions in a caring and individualised way responding to patient need facilitated implementation. Lack of staff time and understanding of interventions, as well as high levels of staff turnover, were barriers to implementation.

**Conclusion:**

This review highlights a striking lack of research and therefore evidence base for the use of psychosocial interventions to reduce distress in this vulnerable population, despite current healthcare guidelines. More research is needed to understand which psychosocial interventions can reduce distress and improve wellbeing on inpatient mental health dementia wards, and how interventions should be delivered, to establish clinical and cost effectiveness and minimise staff burden.

**Supplementary Information:**

The online version contains supplementary material available at 10.1186/s12877-024-04965-8.

## Background

The already high prevalence of dementia and the significant predicted increase in those diagnosed with the condition in the coming decades has been widely documented [1]. Behavioural and psychological symptoms of dementia, which can include agitation, anxiety, depression, sleep disturbances, hallucinations, apathy, and disinhibition, are experienced by 80% of people with dementia in the UK [2]. Throughout this paper we refer to these as distress or distress behaviours, using preferred language by people with dementia reflecting that distress can be caused by symptoms of dementia and/or be an expression of unmet needs [3]. 

Inpatient mental health dementia wards, also known as psychiatric wards, provide care for people with dementia experiencing acute levels of distress that is putting their safety or the safety of others at risk [4, 5]. The aim of the mental health admission is to assess and treat the crisis, including distress behaviours. In the UK, people are often detained using the provisions of the Mental Health Act 2007 meaning they can be treated without their consent, and admission often follows a breakdown of care in the home or care home, which can be traumatic for the person with dementia and their family caregiver(s) [6, 7]. Caring for this population is complex as many have multiple long term conditions, and may need palliative care as they come to end of life [5, 8]. A recent systematic review of the characteristics and outcomes of patients on these wards internationally highlights that little is known about current standards of practice and how best to deliver care in this setting [5]. 

The National Institute for Health and Care Excellence (NICE), who provide evidence-based recommendations for care in the UK, emphasise that psychosocial interventions, also described as nonpharmacological interventions, should be the first line of treatment for distress behaviours in dementia care [9]. NICE defines psychosocial interventions as interventions that require specific competencies for delivery, are supported by relevant training and supervision, and provide an enhanced level of intervention [10]. 

Increasingly such interventions are manualised and their effectiveness to reduce distress and support wellbeing for people with dementia in residential care settings has been tested [11–13]. However, there are still significant gaps in dementia care research and practice [14]. In particular, little is known about what psychosocial interventions have been delivered in inpatient mental health dementia care, and whether these were helpful. This is concerning as these wards provide specialist care for those who cannot be safely cared for in residential care, and pharmacological interventions, such as antipsychotic medication, are frequently used to manage distress with concomitant increase in risk of falls, strokes and death [4, 15]. Further understanding of the current research on inpatient mental health dementia wards, including which psychosocial interventions have been delivered and how, positive and negative patient outcomes, and the facilitators and barriers to implementation, is needed to inform future research and practice.

To understand the current literature on this topic, a systematic review of psychosocial interventions in inpatient mental health dementia care was conducted. Based on expert-by-experience and stakeholder feedback, the review focussed on interventions that aim to reduce distress or improve wellbeing for people with dementia, as this is the primary focus of the admission. The following review questions were established:

- What are the characteristics of psychosocial interventions designed to improve wellbeing and reduce distress for patients within inpatient mental health dementia wards?
- What are the positive and negative outcomes for patients receiving these interventions?
- What are the facilitators and barriers to successful implementation?

## Methods

This systematic review follows the PRISMA reporting guidelines, and is registered on PROSPERO (CRD42023429983) [16]. 

### Search strategy

A systematic search of the databases MEDLINE, CINAHL, PsycINFO, and Psychology and Behavioural Sciences Collection was conducted on the EBSCOhost platform, with an additional search on Scopus. Searches were piloted and performed in May 2023. The search strategy was developed with a librarian experienced in systematic reviews and conducted by NT. Reference lists of included studies were examined, and backward and forward citation searches conducted on Google Scholar.

Search terms were: (old* OR elder* OR geriatric* OR senior*) AND ((Psychiatr* OR psychogeriatric* OR “mental health”) N2 (inpatient* OR ward* OR unit* OR acute)) AND (dementia OR alzheimer* OR “cognitive impairment” OR “memory loss”) AND (psychosocial OR psychological OR psychotherapy OR mental health intervention OR nonpharmacological OR person-centred).

Search results were exported to an online software, Rayyan, for screening [17]. All titles and abstracts were screened by NT, with one third of results independently reviewed by EW. Where there was uncertainty, the full text was retrieved. Screening of the full texts was conducted by NT, with one third independently reviewed by EW. All reasons for exclusion were recorded. At both stages of screening, discrepancies were resolved between the two authors following discussion. Where additional information was required to inform inclusion decisions, authors were contacted via email.

Quality assessment of all included articles was conducted by NT using the Mixed Methods Appraisal Tool (MMAT), with one third conducted independently by EW [18]. This tool is not designed to give a score or inform inclusion and exclusion decisions, but provides a framework for assessing qualitative, quantitative and mixed methods studies. One article included authors of this current review (NT, HOM, and BRU) and so was assessed by EW to minimise bias. In case of uncertainty or discrepancies, decisions were discussed between NT and EW to reach consensus without the need to involve a third reviewer.

### Inclusion and exclusion criteria

Inclusion and exclusion criteria were developed and outlined using an adapted PICOS framework (see Supplementary File 1 for justification for the criteria):

- Population: Intervention actively involves patients with dementia, with a diagnosis from a diagnostic criteria or from a clinician. Studies where results for patients with a formal diagnosis cannot be separated from those with other cognitive impairments or other mental health diagnoses were not included.
- Intervention: Psychosocial intervention, using the NICE definition: requires specific competencies for delivery, is supported by relevant training and supervision, and provides an enhanced level of intervention [10]. An additional definition for psychosocial interventions was helpful in clarifying inclusion, in particular for aspects relating to the aims of the intervention [19].
- Context: Mental health or psychiatric ward providing specialist inpatient care for people with dementia in any country. Studies where results for inpatients and community patients cannot be separated were not included.
- Outcome: Outcomes related to reduced distress or improved wellbeing for the person with dementia. Outcomes must be measured using a standardised questionnaire, or where qualitative data or researcher-designed tool is used, the measurement tool must be published and clearly described to enable quality assessment.
- Study type: Presenting novel findings of any design, conducted internationally, published in a peer-reviewed journal in English. No restrictions for date of publication were given.

### Data extraction and synthesis

Data were extracted to a bespoke data extraction form by NT, checked by EW, recording: author(s); date; country; setting; study design; study participants (include dementia stage and type); aims; intervention (dosage, frequency, duration, mode of delivery); interventionist (training); measurement tools (frequency of use); main findings (positive and negative outcomes); and facilitators and barriers to implementation (who reported these). Due to anticipated heterogeneity of interventions, a narrative synthesis was conducted using the tabulation to synthesise data in relation to the stated research questions, following the guidance of Popay et al. [20]. Facilitators and barriers to implementation were coded using inductive coding, and grouped into themes using thematic analysis [21]. Based on a previous scoping of the literature, interventions were grouped by type of intervention. Where there were two or more studies looking at a similar intervention these were combined to create a new category. Where studies reported outcomes not relating to people with dementia, for example for staff or family members, data were not extracted as this is outside the scope of the review questions.

## Results

**Fig. 1:**
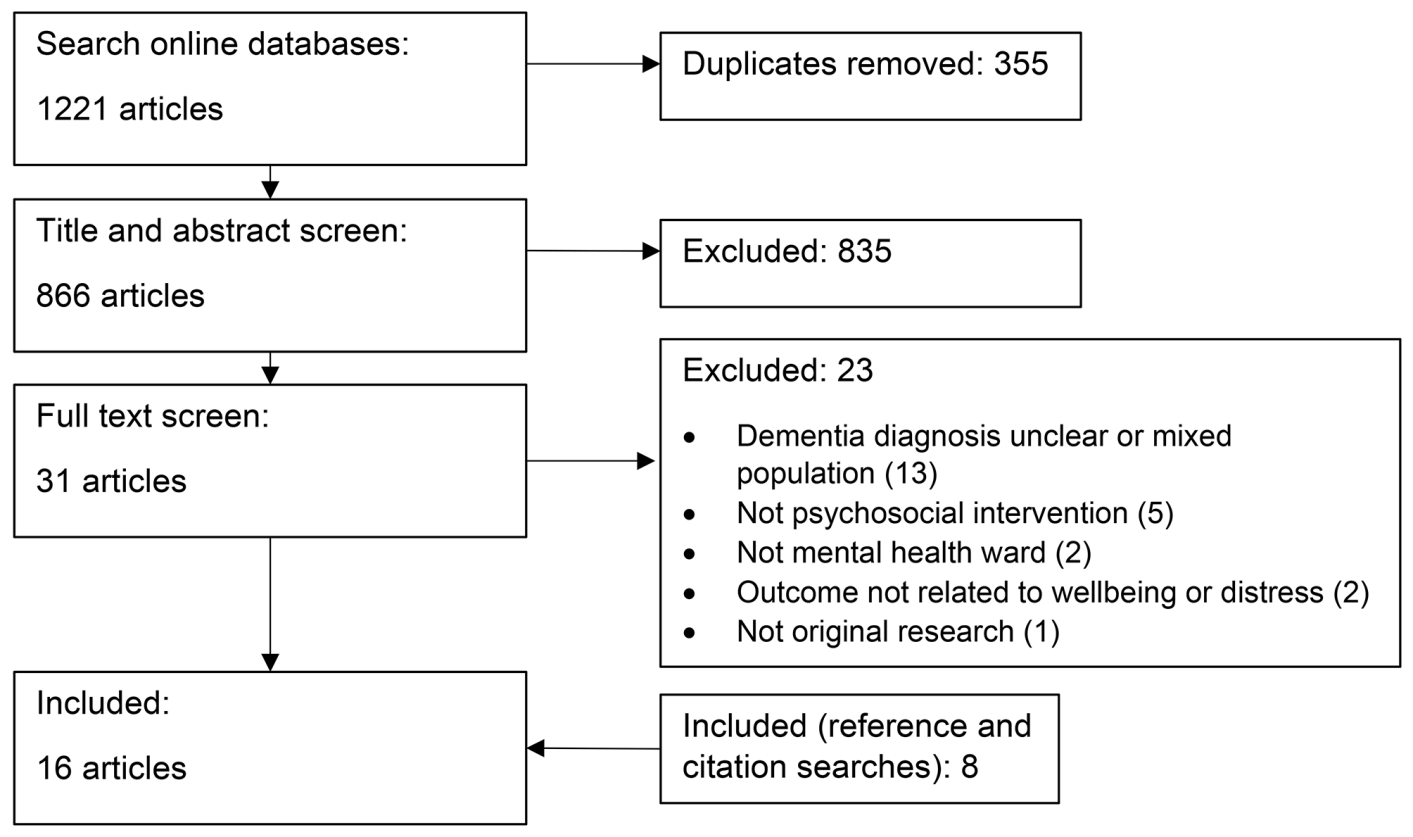
PRISMA diagram of study selection procedure

The online searches retrieved 1221 articles. After removal of 355 duplicates, 866 titles and abstracts were screened. Of these, 835 articles were excluded, with full texts retrieved for 31 articles. Eight articles met the inclusion criteria (see Figure 1 for exclusion reasons). An additional eight articles were included from searching reference lists and citation searches of included articles. A total of 16 articles, involving 16 separate studies, are included in this review.

**Table 1:** Study key characteristics

Author(s), date; country	Setting	Study Design (control where applicable)	Study participants (dementia type where reported)	Aims	Intervention: dosage; frequency; duration; group/individual	Interventionist (training)	Measurement tools (frequency of use)	Main findings (unintended outcomes)
**MUSIC THERAPY INTERVENTIONS**								
Gold, 2014; United Kingdom	1 NHS inpatient unit for advanced stage dementia	Service audit	9 people with advanced dementia; displaying distress behaviours	Increase social connection; increase positive moods and behaviours; reduce negative moods and behaviours	45–60 min; 1 x week; 4 months; group	Accredited music therapist, healthcare assistant supporting	Analysis of ward staff care notes (day of intervention and control)	Increase in positive and decrease in negative moods and behaviours reported for 8/9 patients. No correlation to severity of dementia. (negative impact for 1 patient)
Melhuish, 2013; United Kingdom	1 NHS dementia assessment unit	Pilot project evaluation	22 people with severe dementia	Improve wellbeing, engagement, relationships	1 h; 1 x week; 10 weeks; group	Accredited music therapist, support from ward staff	Analysis of session notes (post each session)	Positive impact on wellbeing, relationship and engagement (on some occasions increased anxiety or agitation during the group); attendance increased over time
Suzuki et al., 2004; Japan	1 dementia special care unit of a geriatric hospital	Case-controlled study (active control)	23 people with dementia; 8 males (12 AD; 11 VD)	Changes in cognition and behaviour, reduction of stress hormone levels	1 h; 2 x week; 8 weeks; group	3 music therapists and 3 nurses (training not stated)	MMSE; N type Mental States Scale and N type ADL; Multidimensional Observation Scale for Elderly Subjects (Pre and 1-week post study period). Pre and post session 1, 8 and 16: Salivary chromogranin A (CgA)	Reduced stress biomarkers, continued to fall throughout study period; reduction in irritability; short term improvement in language, no change in overall cognition
Thomspon et al., 2023; United Kingdom	2 NHS inpatient mental health dementia units	Mixed methods, retrospective, observational study	37 people with dementia	Evaluate impact on distress behaviours	1 h; 1 x week; 1 year; group	Accredited music therapist	Routinely collected ward data (day of intervention and control)	Reduction in staff reported incidents of disruptive and aggressive behaviour on days with in-person music therapy
Thornley, Hirjee and Vasudev, 2016; Canada	1 acute inpatient psychiatric unit	Pilot randomised controlled trial (active control)	16 people with moderate to severe dementia; displaying distressing behaviours and agitation (11 AD, 3 VD, 2 Dementia with Lewy Bodies)	Establish feasibility and acceptability, reduce behavioural and psychological symptoms of dementia	1 h; 2 x week; 4 weeks; individual	Accredited music therapist	Neuropsychiatric Inventory-Caregivers; Cohen-Mansfield Agitation Inventory (baseline and weekly (up to 24 h post intervention) for 4 weeks)	No reduction in agitation, distress behaviours, or difference to active control
**MULTISENSORY INTERVENTIONS**								
Hope, 1998; United Kingdom	Department of old age psychiatry	Mixed-methods study	29 people with dementia; 8 male	Response to multisensory equipment; short and medium term influences on behaviour	As needed; individual	Ward staff (training as needed)	Qualitative behavioural observations; response to equipment (Likert scale); ‘Interact’ scale (frequency and changes in behaviour); pulse rate measure (pre and post intervention)	Majority increased positive mood, relaxation and interactive behaviour; no effect on behaviour post session; no change to heart rate (minority increased agitation)
Mitchell et al., 2015; USA	1 geriatric psychiatric unit	Repeated measures design	13 people with dementia; mild to moderate agitation; 4 male	Reduction in mild to moderate agitation	15–30 min; single use, with repeat visits allowed after 48 h; individual	Staff nurse (training not stated)	PAS; PRN medication post-intervention (pre and post intervention)	Significant reduction in agitation post intervention and 1-hour follow-up, except for aggression subscale
Spaull, Leach and Frampton, 1998; United Kingdom	1 male continuing care ward of a psychiatric unit	Modified single case design	4 people with advanced dementia; 3 displaying behavioural disturbances; all male (2 AD, 2 multi-infarct dementia)	Changes in immediate behaviour, adaptive functioning and wellbeing	20 min; 3 x week; 4 weeks; individual	Occupational therapist (training not stated)	Modified Behaviour Rating Scale and Dementia Care Mapping (pre and post intervention) Short Form Adaptive Behaviour Scale (pre and post study period)	Increased interactive behaviour during sessions, no lasting effect; reduction in unadaptive behaviour post session; no change in wellbeing
Staal et al., 2007; USA	1 geriatric psychiatric unit	Randomised controlled trial (active control)	24 people with moderate to severe dementia; displaying behavioural disturbances; 8 male	Reduction in agitation and apathy, improvement in ADLs	25–30 min; 6 sessions; frequency not stated; individual	Not stated	Global Deterioration Scale; PAS; Multi-level Assessment Instrument - physical health subscale; Scale for the Assessment of Negative Symptoms in Alzheimer’s Disease; Katz Index of ADL; Refined ADL Assessment Scale; MMSE; prescribed antipsychotic medication (pre and post study period)	Improved independence in ADL; reduction in apathy and agitation; no change in medication
**MASSAGE INTERVENTIONS**								
Schaub et al., 2018; Switzerland	1 specialised geriatric psychiatry service	Randomised controlled trial (standard care)	40 people with dementia; experiencing agitation; 20 male	Reduce agitation and biological markers for stress	Hand massage: 16–20 min; 7 massages over 3 weeks; individual	11 nurses and 3 care assistants (2 h group training)	Cohen-Mansfield Agitation Inventory, Salivary Cortisol, Alpha-amylase (before, during (Cohen-Mansfield Inventory only) and after 1st, 4th and 7th massage)	Non-significant reduction in stress biomarkers following repeated sessions; no difference in agitation, agitation increased towards end of afternoon for both groups
Suzuki et al., 2010; Japan	1 specialist dementia unit	Controlled trial (standard care)	28 people with dementia (24 AD, 4 cerebrovascular dementia)	Changes in physical and mental function, behavioural and psychological symptoms of dementia, and stress levels	Tactile massage therapy: 30 min; 5 x week; 6 weeks; individual	Ward nurses (2-day tactile massage training with refresher)	MMSE; Gottfried-Brane-Steen Scale; Behaviour Pathology in Alzheimer’s Disease Rating Scale (Pre and post study period). Salivary CgA (Immediately before and after first and last massage session)	No significant change in cognition; reduction in emotional function in IG and intellectual function in CG; significant improvement in aggression in IG; significant reduction in stress biomarkers in IG
**MULTICOMPONENT INTERVENTIONS**								
Arno and Frank, 1994; not stated	1 female inpatient psychiatric dementia unit	Case study	8 people with moderate or advanced dementia; all female	Improve quality of life	Structured movement and sensory stimulation: 90 min; 1 x week; 9 weeks; group	Nurse leader and coleader (training not stated)	Non standardised group evaluation (post intervention)	Development of group cohesion and norms, apparent reduction in anxiety and changes in social interactions, maintenance of functional abilities. No lasting effect.
Pitkänen et al., 2019; Finland	1 acute psychogeriatric unit	Bench-mark controlled trial (standard care)	175 people with dementia; 79 male (125 AD, 19 VD, 7 other, 18 not specified)	Measure impact on neuropsychiatric symptoms, ADLs and use of psychotropic medications	Music intervention and physical exercise: biweekly music groups and physical exercise group; 45 min; daily individual music and exercise activities; 30 min; 2 years; group and individual	Ward staff (t8 training sessions over 5.5 days)	Neuropsychiatric Inventory, MMSE, Barthel Index, Alzheimer’s Disease Cooperative Study-ADL; demographic information; medication use (on admission and before discharge)	No significant differences between groups. Potential reduction in anxiety in IG compared to CG, but sleep and nighttime behaviour improved less in IG than CG
**TECHNOLOGY-BASED INTERVENTIONS**								
Hung et al., 2018; Canada	1 older adult mental health unit	Mixed methods study	4 people with dementia; displaying responsive behaviours; 1 male (AD, VD, Parkinson’s dementia)	Feasibility and acceptability in this setting, prevent responsive behaviours, engage in meaningful activities, and improve quality of care	iPad simulated presence therapy intervention: individual	Ward staff (training not stated)	Observation and video recordings of care interactions (during intervention)	Positive changes in mood and reduction in anxiety, reduced resistance and increased active involvement in care interventions (video with too many people causing negative response)
Vahia et al., 2017; USA	1 Senior Behaviour Health Inpatient Unit	Longitudinal, open label study	36 people with dementia; varying stages of cognitive impairment; 14 male	Reduce agitation, feasibility of intervention with this population, and relationship between severity of dementia and app use	Supervised use of tablets with range of patient-preferred apps installed: as needed; individual	Nurses and study volunteers (training not stated)	App usage and length of engagement; reduction in agitation on a scale of 1–5 (post intervention)	All patients tolerated tablet use; median use 3x during stay; patients with mild cognitive impairment used more complex apps for longer than those with severe impairment, and staff reported greater reduction in agitation; no adverse events
**EXERCISE INTERVENTIONS**								
Fleiner et al., 2017; Germany	3 specialised dementia care units in department of geriatric psychiatry	Randomised controlled trial (active control)	70 people with moderate dementia; 33 male (26 AD, 9 VD, 32 mixed type, 3 other)	Reduction of neuropsychiatric signs and symptoms, and use of psychotropic medication	Structured exercise intervention: 4 × 20 min sessions; 3 x week; 2 weeks; group	Not stated	Alzheimer’s Disease cooperative study-clinical global impression of change; Neuropsychiatric Inventory; Cohen-Mansfield Agitation Inventory; dosage of antipsychotic medication (Pre and post study period)	Reduction neuropsychiatric signs and symptoms for both groups, IG significantly greater reduction in agitation, lability and verbal aggression, but not physical aggression; no relation to dementia type; no difference in medication dosage

*AD = Alzheimer’s Disease; VD = Vascular Dementia; IG = Intervention Group; CG = Control Group; MMSE– Mini-Mental State Examination; ADL = Activities of Daily Living; PAS = Pittsburgh Agitation Scale; min = minute; h = hour*

### Study characteristics

Study characteristics are displayed in Table 1. Half of the studies were conducted in the United Kingdom (5 studies) and the United States of America (3 studies), while two took place in Canada and Japan, and one in Finland, Switzerland and Germany respectively. It was not possible to clarify where one study was conducted [22]. Articles were published between 1998 and 2023. Methods used included randomised controlled trials [23–26], non-randomised trials [27–29], quantitative descriptive methods [22, 30–32], mixed methods [33, 34], and qualitative studies [35–37]. Outcomes were measured using standardised quantitative tools [23–31], non-standardised quantitative tools [22, 32, 33], biophysiological measures such as pulse and saliva samples [23, 27, 29, 33], qualitative data collection [33–37], and routinely collected ward data [24, 26, 28, 30, 34]. 

The majority of studies investigated a psychosocial intervention on one inpatient mental health ward, while one looked at two wards [34], and another included three wards [26]. There was a combined total of 538 participants across studies, ranging from four to 175 participants. For details of participant characteristics, see Table 1.

### Quality Appraisal

Results from the MMAT showed the varying quality of the included studies, with an average (both mean and mode) of four out of seven criteria met across all studies, ranging from one to seven (l Supplementary File 2). The two screening questions applied to all studies were not consistently met, with two studies not clearly stating the research questions [30, 36], and four not clearly demonstrating they collected appropriate data to answer research questions [22, 30, 35, 36]. However, due to the small number of studies meeting inclusion criteria, these are included in the synthesis.

### Intervention characteristics

The types of interventions delivered included music therapy (five studies [25, 29, 34, 36, 37]), multisensory interventions (four studies [24, 30, 31, 33]), multicomponent interventions (two studies [22, 28]), technology-based interventions, such as using applications or watching videos on tablets, (two studies [32, 35]), massage interventions (two studies [23, 27]), and physical exercise (one study [26]),.

Stated aims of the interventions were wide ranging, with the majority (15 studies) citing multiple aims. The most common aims related to reducing distress behaviours (also referred to as immediate, responsive or negative behaviours) [29, 31, 33–36], reducing agitation [23, 24, 30, 32], reducing neuropsychiatric or behavioural and psychological symptoms of dementia [25–28], and improving activities of daily living and functional abilities [22, 24, 27, 28]. Other aims included the feasibility and acceptability of the intervention in relation to the population [32] and the environment [25, 35], improving mood and wellbeing [31, 36, 37], quality of life [22], quality of care [35], social interaction and engagement [22, 35–37], and cognition [27, 29], as well as reducing apathy [24], use of psychotropic medication [26, 28], and stress (including biomarkers for stress) [23, 27, 29]. 

Intervention delivery was led by ward staff in eight studies (including nurses, care assistants and volunteers) [22, 23, 27, 28, 30, 32, 33, 35], four of which specified that training was provided [23, 27, 28, 33]. Training included ad hoc delivery to individuals as required, and group training ranging from two hours to 5.5 days. In five studies the intervention was delivered by a music therapist [25, 29, 34, 36, 37], with all but one specifying that the therapist was accredited with the relevant healthcare board [29], and three of which stated that ward staff supported in the sessions [29, 36, 37]. An occupational therapist delivered the intervention in one study, although training was not stated [31]. The interventionist was not specified in two studies [24, 26]. 

Most studies delivered the intervention on an individual basis [23–25, 27, 30–33, 35], while six interventions were delivered on a group basis [22, 26, 29, 34, 36, 37]. One multicomponent intervention study included both group and individual sessions [28]. 

Intervention frequency ranged from weekly [22, 34, 36, 37], to twice a week [23, 25, 29], and more than twice a week [26–28, 31], with three interventions conducted as needed [30, 32, 33]. The dosage (i.e. length of each session) was reported by 13 studies, with five lasting up to 30 min [23, 24, 27, 30, 31], six between 31and 60 min [25, 28, 29, 34, 36, 37], and two over 60 min [22, 26]. The duration of the intervention period was reported by 11 studies with the majority running for up to four weeks [23, 25, 26, 31], or five to ten weeks [22, 27, 29, 37], and others lasting four months [36], one year [34], and two years [28]. 

### Intervention outcomes

#### Music therapy intervention outcomes

A reduction in agitation and distress behaviours, and increase in positive moods and behaviours, was reported in four of the five music therapy interventions on days when the intervention was delivered [29, 34, 36, 37], one of which also reporting a reduction in biomarkers for stress [29]. Two of these studies found that a minority of participants displayed increased frustration or agitation during group music therapy sessions [36, 37]. One study suggested that this could be a response from the participant to being drawn out of passivity [36], and another that the open nature of the group enabled participants to leave if they chose to [37]. However, one study reported no reduction in agitation or distress behaviours when music therapy was compared to an active control, though the groups were not comparable at baseline and it was not clear whether participants had adhered to the assigned intervention [25]. 

#### Multisensory intervention outcomes

All of the multisensory interventions reported short term positive outcomes relating to reduction in agitation and distress behaviours and increases in positive moods and interactive behaviours during sessions [24, 30, 31, 33], with one study reporting this lasted up to one hour post intervention [30]. Additional reported outcomes were improved independence in activities of daily living [24], and reduction in apathy [24]. No change was reported for aggressive behaviours [30], wellbeing [31], heart rate [33], and prescribed medication [24]. 

#### Multicomponent, massage, technology-based and physical exercise intervention outcomes

Outcomes for massage interventions were inconclusive. Two studies reported a reduction in biomarkers for stress following massage [23, 27], with one study, which accounted for confounding factors in the analysis, reaching statistical significance [27]. One study found no change in cognition but a significant reduction in aggression [27], while one found no difference in agitation [23], although authors suggest this could be because scores for agitation were low at baseline, and quality assessment showed that reported outcome data were not complete.

For multicomponent interventions, reported outcomes were conflicting. One study reported short term development of group cohesion, reduction in anxiety and changes in social interactions following weekly movement and sensory stimulation groups [22]. However, another found no significant differences between the intervention group, receiving group and individual music and physical exercise, and a control group of previous patient cohorts receiving standard care, although data suggested a reduction in anxiety and worsening of sleep and nighttime behaviour [28]. This study was a randomised controlled trial with a large number of participants, but it was not clear whether the intervention was administered as intended and confounding factors were not accounted for in the design and analysis.

Reported findings for technology-based interventions suggest positive changes in mood and reduction in anxiety, agitation and resistance to care [32, 35]. However, one intervention using simulated presence through recorded videos of family members, found that videos with too many people could cause a negative response [35]. 

Finally, a study of a physical exercise intervention found a significant reduction in agitation, lability and verbal aggression in the intervention group, but no reduction in physical aggression or prescribed medication [26]. This study also reported that participants did not adhere to the assigned intervention.

### Facilitators and barriers to implementation

All except one [31] of the included studies reported facilitators and/or barriers to implementation of the psychosocial intervention in the ward setting. Most were reported by the researchers, but some were reflections from staff, with one [37] also including feedback from family members. Inductive coding of reported facilitators and barriers led to the emergence of three themes: factors relating to the interventionist, factors relating to the intervention, and factors relating to the ward environment.

#### Factors relating to the interventionist

Researchers, staff and family members reported that staff support and understanding of the intervention, and delivering it with a caring approach enabling patients to express themselves and interact as they were able in the moment, facilitated implementation [22, 32, 34, 35, 37]. Researchers reported that the provision of supervision supported understanding [32], and family members and staff stated that observing the positive effects of the intervention with opportunities for positive interactions with patients were additional facilitators [37]. One study reported that nurse initiation of the intervention without referral to more senior staff members for approval supported implementation [30]. Barriers to implementation support these findings, with five studies, two of which reported staff feedback, stating that lack of understanding, scepticism of the intervention, and resistance to having close relationships with patients, were barriers to implementation and effectiveness [23, 27, 34, 35, 37]. This included staff reported fears about using equipment incorrectly or causing negative effects [33]. 

#### Factors relating to the intervention

Seven studies, two reporting staff responses, stated that the ability to individualise the intervention to patient preference, ability and the patient’s culture facilitated implementation [22, 24, 29, 30, 32, 34, 37]. In addition, researchers and staff in four studies reported that utilising nonverbal methods of communication, such as touch and music, enabled emotional expression, increased engagement and attention, and helped deepen the relationship between participants [27, 29, 34, 37]. Other facilitating elements reported by researchers were safe, easy and accessible delivery [35], and gradually increasing the length of the intervention [24], while staff reported using good quality video and audio materials as supporting factors [35]. Factors reported by researchers as barriers to implementation of interventions were overstimulation for the person with dementia [35], and not being tailored to the cognitive abilities of the individual, such as concentration, with one study reporting that this was particularly evident towards the beginning of the hospital stay [28, 35]. Additionally, how interventions were introduced to the wards could be a barrier to implementation including a lack of clear plans for implementation in the design [33], and introducing multiple interventions simultaneously [28]. 

#### Factors relating to the ward environment

Elements relating to the ward environment were cited as both facilitators and barriers to implementation. Enabling factors reported by staff and researchers were the ability to create a calm space on the ward [22, 35], regular intervention delivery, which may enable a trusting relationship to be established between patient and interventionist [23, 26, 27, 29], and timing the intervention around patients’ needs and ward routines [23, 26, 27]. Inhibiting factors reported by researchers were rigid timing of intervention delivery to fit around ward routines [23], a clinical focus on behaviour rather than mood on the ward [36], lack of staff time to support and deliver interventions [28, 37], high levels of staff turnover, and not having regular access to an appropriate space [33]. In addition, one study suggested that patients being in late stages of dementia could be a barrier to engaging in interventions [25]. However, in this study, treatment groups were not comparable at baseline and the intervention did not appear to be individualised to the patient [25]. 

## Discussion

This review provides a systematic, narrative analysis of psychosocial interventions reported on inpatient mental health dementia wards, the outcomes for patients, staff and families, and the factors influencing implementation. The 16 included studies were small and of varying quality, but suggest that psychosocial interventions may help reduce distress experienced by people with dementia on these wards. The lack of good quality research is particularly striking given that NICE guidelines call for psychosocial interventions as the first line of treatment for people with dementia experiencing distress, and these wards provide care for those experiencing the highest levels of distress in our communities [4, 9]. There is therefore a critical need for more research in this area, as reported in previous systematic reviews [5]. 

The research included in this review was of varying methodological quality and mostly in early stages of research development, with small samples and using single sites. This is reflected in the results of the MMAT with studies not consistently stating the research questions or demonstrating data collected were appropriate to answer research questions. This limits the comparability of results between studies, and the generalisability of findings to other settings. In addition, justification for the chosen intervention, the way it was delivered, and the theory for how and why it is expected to reduce distress and improve wellbeing, was poorly reported.

Overall findings suggest that psychosocial interventions, in particular music therapy and multisensory interventions, may be helpful in reducing distress and potentially improving wellbeing, although findings were not consistent. This is supported by a reduction in stress biomarkers in some included studies, suggesting interventions could have a biophysiological impact on people with dementia which enables a reduction in distress [23, 27, 29]. However, the need for careful intervention design and delivery were highlighted by reported negative outcomes including worsening sleep behaviour and overstimulation [28, 35]. The potential challenges of implementing psychosocial interventions in this ward environment were shown. In particular, most interventions relied on staff for delivery, but lack of staff time and understanding of the intervention, and high levels of staff turnover, were barriers to implementation.

Findings from this review suggest that psychosocial interventions should be mainly nonverbal, person-centred, culturally sensitive, and delivered flexibly by a trained and skilled interventionist who is able to respond and regulate arousal in the moment. They should also minimise reliance on staff to deliver them and provide adequate training for staff to understand the potential benefits of the intervention. This has implications for policy, with psychosocial interventions reviewed against these criteria to increase their usefulness and helpfulness in reducing distress for people with dementia on mental health wards. In particular, policies should support training for staff to deliver specific psychosocial interventions, with accompanying funding for this post above the current staffing on wards, and the inclusion of mandatory training on interventions for all staff. Additionally, policies should include the development of standards for dementia friendly ward environments to ensure that psychosocial interventions can be implemented, including having private spaces available to deliver individual and small group interventions.

However, included studies do not provide evidence for which interventions should be delivered, how and when to deliver them, the support needed for implementation, and how this links with the wider care plan and support for the individual. There was also a lack of evidence for the involvement and impact for family members, with only one [37] study mentioning their involvement. Future research should consult current guidelines on developing and evaluating complex interventions, such as those from the Medical Research Council [38]. Involving people with lived experience, such as staff, family members and patients, in the design of interventions and studies will be crucial to supporting feasibility of delivery and helpfulness [39, 40]. Once these factors are identified, multi-site, randomised and masked studies are needed to establish clinical and cost effectiveness. Outcomes should include the effectiveness of interventions to reduce distress, with definitions and outcome measures agreed with staff, patients and family members; the cost-effectiveness of the intervention; the impact on staff time, care delivery and the ward environment; the impact on patient, staff and family member wellbeing; the impact on patient length of stay, including communication with the discharge destination; and the impact on use of as-needed (pro re nata) and prescribed medication.

Limitations of this review include the use of a second reviewer for only one third of the titles and abstracts and full text articles during the screening process meaning it is possible that articles were wrongly excluded during screening. Only articles written in English were included due to resource limitations, and articles not published in peer-review journals were excluded, potentially missing ward-based evaluations and audits. Additionally, the varied language used to describe inpatient mental health wards internationally further complicated the screening process. Studies specifying they took place on hospital wards and focused on distress behaviours in dementia were included, and any uncertainties were discussed with the team. Due to the small number of studies expected to meet the criteria a time limit was not used so some studies may not reflect current practice.

## Conclusion

Further research is required to increase our understanding of whether specific psychosocial interventions can help reduce distress and improve wellbeing for people with dementia on inpatient mental health dementia wards, and how these should be delivered. This can enable the development of cost-effective toolkits and protocols for psychosocial interventions that are feasible to deliver with limited resource and have been shown to reduce distress and improve wellbeing on inpatient mental health dementia wards.

### Electronic supplementary material

Below is the link to the electronic supplementary material.

Supplementary Material 1

Supplementary Material 2

## Data Availability

All data generated or analysed during this study are included in this published article [and its supplementary information files].

## Abbreviations

EW: Emma Wolverson
MMAT: Mixed Methods Appraisal Tool
NICE: National Institute for Health and Care Excellence
NT: Naomi Thompson
PICOS: Participant, Intervention, Control, Outcome, Study Type
