# Supplementary material for: Characteristics, outcomes, facilitators and barriers for psychosocial interventions on inpatient mental health dementia wards: a systematic review
Source: BMC Geriatr. 2024 Apr 23;24:364. doi: 10.1186/s12877-024-04965-8 (PMC11040912; doi:10.1186/s12877-024-04965-8)
Supplement: Supplementary file 1 — Supplementary Material 1 [file 12877_2024_4965_MOESM1_ESM.docx]

**Additional file 2** Results from MMAT Quality Appraisal Tool.

| Qualitative studies | | | | | | | |
| --- | --- | --- | --- | --- | --- | --- | --- |
| Study | Are there clear research questions? | Do the collected data allow to address the research questions? | Is the qualitative approach appropriate to answer the research questions? | Are the qualitative data collection methods adequate to address the research question? | Are the findings adequately derived from the data? | Is the interpretation of results sufficiently substantiated by data? | Is there coherence between data sources, collection, analysis and interpretation? |
| Hung et al., 2018 | Y | C | Y | C | N | Y | Y |
| Gold, 2014 | N | C | Y | Y | Y | Y | Y |
| Melhuish, 2013 | Y | Y | Y | Y | C | Y | Y |
| Quantitative Randomised Controlled Trials | | | | | | | |
| Study | Are there clear research questions? | Do the collected data allow to address the research questions? | Is randomisation appropriately performed? | Are the groups comparable at baseline? | Are there complete outcome data? | Are outcome assessors blinded to the intervention provided? | Did the participants adhere to the assigned intervention? |
| Staal et al., 2007 | Y | Y | C | N | C | C | C |
| Schaub et al., 2018 | Y | Y | Y | Y | N | C | C |
| Thornley et al., 2016 | Y | Y | Y | N | C | Y | C |
| Fleiner et al., 2017 | Y | Y | Y | Y | Y | Y | N |
| Quantitative non-randomised | | | | | | | |
| Study | Are there clear research questions? | Do the collected data allow to address the research questions? | Are the participants representative of the target population? | Are measurements appropriate regarding both the outcome and intervention (or exposure)? | Are there complete outcome data? | Are the confounders accounted for in the design and analysis? | During the study period, is the intervention administered (or exposure occurred) as intended? |
| Pitkänen et al., 2019 | Y | Y | Y | Y | Y | N | C |
| Suzuki et al., 2004 | Y | Y | C | Y | N | N | C |
| Suzuki et al., 2010 | Y | Y | C | Y | N | Y | N |
| Quantitative descriptive studies | | | | | | | |
| Study | Are there clear research questions? | Do the collected data allow to address the research questions? | Is the sampling strategy relevant to address the research question? | Is the sample representative of the target population? | Are the measurements appropriate? | Is the risk of nonresponse bias low? | Is the statistical analysis appropriate to answer the research question? |
| Mitchell et al., 2015 | N | C | C | C | Y | C | Y |
| Spaull et al., 1998 | Y | Y | C | C | Y | C | Y |
| Arno and Frank, 1994 | Y | N | C | C | N | N | C |
| Vahia et al., 2017 | Y | Y | Y | Y | Y | N | Y |
| Mixed Methods | | | | | | | |
| Study | Are there clear research questions? | Do the collected data allow to address the research questions? | Is there an adequate rationale for using a mixed methods design to address the research question? | Are the different components of the study effectively integrated to answer the research question? | Are the outputs of the integration of qualitative and quantitative components adequately interpreted? | Are divergences and inconsistencies between quantitative and qualitative results adequately addressed? | Do the different components of the study adhere to the quality criteria of each tradition of the methods involved? |
| Thompson et al., 2023 | Y | Y | Y | Y | Y | Y | Y |
| Hope, 1998 | Y | Y | Y | Y | N | Y | N |

Y = Yes; N = No; C = Can't Tell
