# Supplementary material for: Characteristics, outcomes, facilitators and barriers for psychosocial interventions on inpatient mental health dementia wards: a systematic review
Source: BMC Geriatr. 2024 Apr 23;24:364. doi: 10.1186/s12877-024-04965-8 (PMC11040912; doi:10.1186/s12877-024-04965-8)
Supplement: Supplementary file 2 — Supplementary Material 2 [file 12877_2024_4965_MOESM2_ESM.docx]

**Additional file 1**: Justification for search criteria

| CRITERIA using PICOS | JUSTIFICATION |
| --- | --- |
| Population: Patients with dementia diagnosis | Recipient of the intervention to be a person with a formal diagnosis of any type or stage of dementia. Diagnosis can be from a diagnostic manual or from a relevant clinician. Not to include studies where results for people with dementia cannot be separated from results for people with other cognitive impairments or functional mental health diagnoses; and studies where the intervention focusses on staff or family members. |
| Intervention: Psychosocial intervention as defined by NICE: which requires specific competencies for delivery, is supported by relevant training and supervision, and provides an enhanced level of intervention.(1) | There are increasing calls for psychosocial and nonpharmacological interventions to reduce distress and related behaviours experienced by people with dementia, as highlighted in the recent World Health Organisation’s blueprint for dementia research.(2) There is a need to better understand what research has already been conducted to refine and develop new interventions in this area. |
| Context: Mental health ward providing inpatient care for people with dementia | An inpatient mental health or psychiatric ward or unit where the individual is hospitalised for specialist treatment and support because of their dementia. We recognise there is no standard model of care for this vulnerable population, with some wards providing specialist dementia support, others providing care for people with a range of diagnoses or staying in adult mental health wards. Additionally, specialist dementia wards can have a range of names. We will exclude where the setting is unclear and where there is a mixed community and inpatient sample. |
| Outcome: Outcomes related to reduced distress or improved wellbeing for the person with dementia | The review will focus on reduction of distress or improvement in wellbeing, as this is the focus of the hospital stay for people with dementia on mental health wards, and has been identified as the most important area for research by clinicians and experts-by-experience. Outcomes must be measured using a standardised questionnaire, or where qualitative data or researcher-designed tool is used, the measurement tool must be published and clearly described to enable quality assessment. Interventions focussing on other outcomes, such as cognition or activities of daily living, will be excluded where results cannot be separated. |
| Study type: Presenting novel findings of any design; in any country; any date; English | A systematic review of randomised controlled trials of nonpharmacological interventions to reduce agitation for people with dementia found no studies conducted in inpatient mental health settings.(3) This review will therefore include studies of any design, including qualitative, quantitative, case and service evaluation studies as it is anticipated much research will be in the earlier stages of development. Capturing this data will enable us to synthesise this information. Studies of any date will be included for similar reasons, though the authors recognise that inpatient care has changed over time. Only articles written in English are included as there is no funding to support translation. |

References:

1. National Institute for Health and Care Excellence. Drug use disorders in adults: Quality standard [Internet]. 2012. Available from: www.nice.org.uk/guidance/qs23

2. World Health Organisation. A blueprint for dementia research. 2022.

3. Livingston G, Kelly L, Lewis-Holmes E, Baio G, Morris S, Patel N, et al. Non-pharmacological interventions for agitation in dementia: systematic review of randomised controlled trials. British Journal of Psychiatry [Internet]. 2014 Dec;205(6):436–42. Available from: https://search.ebscohost.com/login.aspx?direct=true&db=pbh&AN=99913155&site=ehost-live
